# Supplementary material for: Comparing P300 flashing paradigms in online typing with language models
Source: PLoS One. 2025 Feb 3;20(2):e0303390. doi: 10.1371/journal.pone.0303390 (PMC11790102; doi:10.1371/journal.pone.0303390)
Supplement: Attachment — Submitted filename: reviewer_response2.docx [file pone.0303390.s002.docx]

**Reviewer #1: Table 2: Replace "Reimann" with "Riemann"**

We have corrected this typo.

**Line 244-255: It is mentioned that "None of these decreases was statistically significant" Later, it is mentioned that "The CBP performance was not significantly different from the full dataset". Were statistical tests carried out within one flashing pattern, comparing its performance over different amounts of training data, or within one amount of training data, comparing different flashing patterns with each other? From the text, it seems that the first strategy was chosen, but to verify the claim whether the flashing pattern has impact on performance, the second strategy should be used.**

We thank the reviewer for this clarification and agree that the analysis they propose is more appropriate. When reducing the training set, the COMB paradigm still produces the highest ITR in offline experiments. This difference remains significant in the 50% case. When reducing the training set to 30%, however, the difference between COMB and CBP is no longer significant. This analysis has been added to the results section.

**Line 256-264: Similar remark. the text tests and discusses whether there is an impact of the classifier of performance within the different flashing patterns, not whether there are differences in performance across flashing patterns within a given classifier. Only the latter strategy contributes to verifying whether the flashing patterns impact performance, which is, to the reviewer's understanding, what is investigated here.**

Since the primary goal of this paper was to explore the performance of the models during online experiments, we believed that the main aim when comparing classifiers offline was to justify the use of LDA in online experiments. However, we recognize the interest in investigating whether the trends in LDA extend to other classifiers. We have therefore adjusted this paragraph to include results comparing flashing paradigms. Overall, the trend of COMB having the best ITR is persistent across classifiers, although it is only significant when using LDA and RF.
